# Clinical utility of the Xpert MRSA assay for early detection of methicillin-resistant *Staphylococcus aureus*

**DOI:** 10.3892/mmr.2012.1121

**Published:** 2012-10-09

**Authors:** AE-CHIN OH, JIN KYUNG LEE, HA NA LEE, YOUNG JUN HONG, YOON HWAN CHANG, SEOK-IL HONG, DONG HO KIM

**Affiliations:** 1 Department of Laboratory Medicine, Korea Cancer Center Hospital, Nowon-gu, Seoul 139-706; 2 Department of Pediatrics, Korea Cancer Center Hospital, Nowon-gu, Seoul 139-706; 3 KIRAMS Radiation Biorepository, Korea Institute of Radiological and Medical Sciences, Nowon-gu, Seoul 139-706; 4 College of Pharmacy, Seoul National University Graduate School, Gwanak-gu, Seoul 151-742, Republic of Korea

**Keywords:** MRSA, Xpert^®^ MRSA, early detection, lower respiratory tract infection

## Abstract

Methicillin-resistant *Staphylococcus aureus* (MRSA) is responsible for many nosocomial and community-acquired infections, resulting in significant morbidity and mortality. A practical way to limit the spread of MRSA is early detection and proper treatment. However, screening culture for MRSA typically requires 2–3 days. The Xpert MRSA assay (Cepheid, Sunnyvale, CA, USA) is a real-time polymerase chain reaction-based assay developed for screening an MRSA-specific DNA sequence within the staphylococcal cassette chromosome in 2 h. Lower respiratory tract specimens, such as transtracheal aspirates (TTAs) and bronchoalveolar lavage fluid (BALF), are commonly obtained from intubated patients. Therefore, using the lower respiratory tract specimens with the Xpert MRSA assay may be a practical tool for patient care. We performed the Xpert MRSA assay on 108 TTA and 21 BALF specimens from 92 patients and compared the results to those obtained by culture. The two assays showed concordant results in 120 (93.0%) cases and discordant results in 9 (7.0%) cases, which were culture-negative and Xpert MRSA-positive. Among the discordant cases, 5 patients developed culture-positive samples 2–15 days after the Xpert MRSA detected MRSA. We conclude that the Xpert MRSA assay is a rapid, sensitive and clinically useful test, particularly for the early detection of MRSA.

## Introduction

*Staphylococcus aureus* is a well-documented opportunistic human pathogen and a major nosocomial pathogen that causes a range of diseases. Infections caused by *S. aureus* range from skin and soft-tissue infections, to pneumonia and bacteremia. Methicillin was introduced as an agent to treat staphylococcal infections in 1959 (1). However, methicillin-resistant *Staphylococcus aureus* (MRSA) strains were identified by 1960. MRSA is responsible for many nosocomial and community-acquired infections and results in significant morbidity and mortality (1,2). It is critical to limit the spread of MRSA in health care settings. A practical way to limit the spread of MRSA is early detection and appropriate treatment of the infection (3). The standard methods used for MRSA detection include the use of oxacillin-salt agar plates, disk-diffusion susceptibility testing and an automated MRSA identification system (i.e., Microscan^®^ and Vitek^®^) (4). However, screening samples for MRSA typically requires 2–3 days (4), and such a delay in diagnosis allows MRSA to spread. Advances in molecular diagnostics have enabled the use of real-time polymerase chain reaction (PCR) for detecting and monitoring organisms in clinical specimens (6). The majority of the molecular tests for MRSA detect the presence of the *mecA* gene, and this gene is also carried by certain methicillin-susceptible *Staphylococcus aureus* (MSSA) and methicillin-resistant coagulase-negative staphylococci (MRCNSs) (7). To avoid a false-positive result, it is necessary to target a gene specific to methicillin-resistant *S. aureus*(7,8). The Xpert MRSA assay (Cepheid) has been developed for screening an MRSA-specific DNA sequence within the staphylococcal cassette chromosome *mec* (SCC*mec*), found only in *S. aureus* and not in other staphylococci (9). This is a real-time PCR-based assay that automates and integrates all the steps of SCC*mec* detection from DNA extraction to detection of the target organism within 2 h. The Xpert MRSA kit has been approved for the screening of MRSA in nasal swab specimens (10). However, there are questions as to whether body sites other than the nose should be sampled for MRSA surveillance (11). Lower-respiratory-tract specimens such as transtracheal aspirates (TTAs) and bronchoalveolar lavage fluid (BALF) are often required in intubated patients and patients from whom suitable respiratory secretions cannot be obtained (12). The Xpert MRSA assay using lower-respiratory tract specimens may be useful for diagnosing respiratory infection by MRSA. The aim of this study was to evaluate and compare the clinical usefulness of the Xpert MRSA assay with culture methods for the early detection of lower respiratory infection by MRSA. We evaluated the speed, reliability and clinical usefulness of the Xpert MRSA assay and analyzed the applicability of this rapid assay in clinical microbiological laboratories.

## Materials and methods

### Specimen selection

Lower-respiratory-tract specimens such as TTA and BALF were obtained from 92 patients who were hospitalized in the Intensive Care Unit of Korea Cancer Center Hospital and sent to our clinical microbiology laboratory for routine culture to diagnose respiratory infection. After the culture was completed, the left-over specimens were anonymized and submitted to the biorepository in our institute (KIRAMS Radiation Biorepository, KRB), with the informed consent of the patients or guardians. For the Xpert MRSA assay, the specimens were distributed from the KRB. This protocol was approved by the institutional review board of the Korea Institute of Radiological and Medical Sciences (K-1111-002-017).

### Culture and identification of MRSA

One swab was obtained from each specimen and streaked onto a blood agar plate and incubated in 5% CO_2_ at 36°C for 24 h. When the colonies were suspected to be those of *S. aureus* on the basis of their morphology, they were tested with a Vitek 2 gram-positive identification card (bioMerieux, Marcy l’Étoile, France) for identification. Methicillin resistance was then determined by measuring the minimum concentration of oxacillin required to inhibit the bacteria using the Vitek broth culture system (bioMerieux).

### Xpert MRSA assay

The Xpert MRSA assay was performed using a GeneXpert^®^ Dx system that carries out the PCR process using an Xpert MRSA cartridge containing PCR reagents to detect SCC*mec*. Specimens for the Xpert MRSA assay were disseminated from the KIRAMS Radiation Biorepository. One swab from each archived specimen was suspended into a tube containing elusion reagents. The elusion tubes, containing guanidinium thiocyanate and surfactants, were vortexed at a high speed for 10 sec. The mixture was added to the ‘S’ chamber of the Xpert MRSA cartridge. A solution of sodium hydroxide (reagent 1) was pipetted into chamber 1, while a second reagent (Tris-buffer, EDTA and surfactants) was pipetted into chamber 2 of the cartridge. The prepared cartridge was inserted into the GeneXpert Dx instrument within 15 min of adding the reagents to the cartridge. The analysis was performed as per the manufacturer’s amplification protocol. PCR reactions are considered invalid when the specimens do not meet the acceptance criteria for sample-processing control (SPC). On the basis of the cut-off value of 30 for the threshold cycle (Ct), the PCR results were interpreted to be positive or negative.

### Comparison of the culture method and the Xpert MRSA assay

We categorized the initial culture results as positive or negative for MRSA. The Xpert MRSA assay results were also categorized as positive and negative. The results of the two assays were compared, and each case was classified as concordant or discordant, according to the agreement between the two results. Medical records were reviewed for the discordant group to evaluate the infection history prior to and following the culture test. The concordant MRSA-infected group and discordant early-detected MRSA group were considered as true positives for MRSA infection. We evaluated the sensitivity, specificity, positive predictive value (PPV), negative predictive value (NPV) and false-positive rate of the Xpert MRSA assay.

## Results

### Comparison of the results of culture and Xpert MRSA assay

Out of 129 specimens (108 TTA and 21 BALF) from 92 patients, the Xpert MRSA assay and culture identified 42 (32.6%) and 32 (24.8%) positive specimens, respectively. The patients (65 males and 27 females, median age of 71 years) had various types of cancer. None of the tested specimens were excluded as they all showed valid PCR reactions. Concordant results between the two assays were observed in 120 (93.0%) specimens and discordant results were observed in 9 (7.0%) specimens. All the culture-positive specimens were also tested positive by the Xpert MRSA assay.

### Clinical characteristics of the concordant and discordant cases

Out of the 120 concordant cases, 33 were positive according to Xpert MRSA and culture, which were categorized as MRSA infected, and 87 were negative according to Xpert MRSA and culture, which were categorized as non-infected. All 9 (7.0%) discordant cases showed negative culture and positive Xpert MRSA results (Table I). We reviewed the medical records of the discordant cases. Five (3.9%) out of the 9 cases showed positive results in the next culture test (categorized as ‘early detection’), and the time interval between the positive Xpert MRSA and the positive culture ranged from 2 to 15 days (mean, 8 days). For the 4 (3.1%) remaining cases, the patients had been infected with MRSA in the previous week (categorized as ‘previous infection’), and the time intervals ranged from 1 to 7 days (mean, 4.5 days) (Table II). As we regarded the concordant MRSA-infected group and the early-detected MRSA group as true-positives, the 4 (3.1%) cases of previous infection were concluded to be false-positive. The sensitivity, specificity, PPV and NPV of the Xpert MRSA assay were 100, 90.7, 78.0 and 100%, respectively.

## Discussion

Since delayed detection of MRSA increases the transmission of MRSA among hospitalized patients, rapid and accurate detection is extremely valuable for infection control in clinical settings (20). Although culture-based methods have been the standard methods used to detect MRSA, 2–3 days are required to obtain results by these methods (5). Real-time PCR-based tests, including the Xpert MRSA assay, have been developed and widely used as the technique is simple, sensitive and takes less than 2 h (13). Since the Xpert MRSA assay has been approved for screening MRSA in nasal swab specimens, we aimed to assess the feasibility of lower-respiratory tract specimens such as TTA and BALF. In this study, all the specimens met the acceptance criteria, which verify adequacy of specimen processing and absence of inhibitors of the PCR reaction. TTA and BALF for routine culture were also optimal for the Xpert MRSA assay, and the rapidity of the technique makes this assay more useful for patient care and infection control, with the potential for a reduction in health care costs (4,21).

The majority of the MRSA screening methods focus on detecting the *mecA* gene. This structural gene is responsible for methicillin resistance via the production of an altered penicillin-binding protein, which maintains staphylococcal cell-wall integrity due to its low affinity to β-lactam antibiotics. The *mecA* gene is present within a transposon-encoded genetic region known as the SCC*mec*(14). If a molecular assay targets only for the *mecA* gene, MSSAs and MRCNSs carrying the *mecA* gene may provide false-positive results (8). False-positive results may be explained by the presence of amplifiable DNA originating from non-viable strains in treated patients, genetic excision within the SCC*mec* region, low sensitivity of the culture, or mixed-culture cocktails of MSSA in the absence of MRSA (10,15–17). Presence of dead MRSA cells yields positive results and accounts for the majority of the false-positive results. The duration for which dead MRSA cells can be detected by PCR will be a notable subject for future studies. Genetic excisions within the SCC*mec* region of the MRSA strains in the absence of a functional *mecA* gene may also yield positive PCR results. Thus, these ‘empty cassette variants’ lead to PCR-positive and culture-negative results. These MSSAs are misidentified as MRSA and in fact lead to false-positive PCR results (11,14,15). However, this error does not occur in the case of the Xpert MRSA assay, as it screens for an MRSA-specific DNA sequence within the SCC*mec*. Discrepant results may be due to the differences in the lower limits of detection of the PCR assay. Rossney *et al* have previously reported that the detection limit of agar cultures is 171 CFU/swab, whereas those for broth cultures and the Xpert MRSA assay are 9 CFU/swab and 58 CFU/swab, respectively (16). Positive results in the case of the non-MRSA infected group were due to the presence of other organisms misidentified as MRSA. Cross-reactions may occur when non-MRSA strains are infected together with MRSA. Another study pointed out that the sensitivity for MRSA strains in mixtures spiked with non-MRSA strains is higher than that for pure MRSA strains (9).

Sputum specimens are often collected from intubated patients, wherein complete elimination of contamination by resident oral bacteria is impossible (12). Specimens obtained from the lower respiratory tract are tested in order to detect the pathogen causing hospital-acquired pneumonia (12). Invasive methods for collecting specimens such as TTA or BALF are indicated when appropriate respiratory secretions cannot be obtained (12).

Our study has a few limitations. Firstly, the culture result was not confirmed for the presence of MRSA by more sensitive techniques. For bacterial culture, we do not use broth enrichment media or selective agar as a routine procedure. Therefore, we implemented the same procedure as our routine culture and compared it with the Xpert MRSA assay. The isolation rate of MRSA with broth enrichment or selective agar is higher than that with agar culture alone, but the technique is not always beneficial to every clinical laboratory due to workload and turnaround time (18). Secondly, the specimens we used have not been clinically approved to date. However, a nasal swab, which is the only approved specimen for MRSA screening using the Xpert MRSA assay, is not always the best specimen. A previous study showed that MRSA nasal colonization was a poor predictor of subsequent MRSA infection. They pointed out that 72.5% of the patients with lower respiratory infection with MRSA and 73.1% of the patients with blood stream infection with MRSA showed negative results for nasal colonization (19). Therefore, an effort to gather appropriate specimens should be made.

In conclusion, the Xpert MRSA assay is a simple, rapid and sensitive technique to detect MRSA and may be beneficial for early detection in a clinical laboratory.

## Figures and Tables

**Table I:** Comparison of the results of culture and Xpert MRSA assay.

	Xpert		
		
	+	−	Total
Culture			
+	33	0	33
−	9	87	96
Total	42	87	129

Xpert, Xpert MRSA assay; +, positive; −, negative; MRSA, methicillin- resistant *Staphylococcus aureus*.

**Table II:** Clinical specifications of the 9 discordant cases which showed positive Xpert MRSA and negative culture.

		Assay results						
					
Case	Gender/age		1st	2nd	3rd	Interval between Xpert(+)and culture(+)^a^	State^b^	MRSA decision^c^
1	M/67	Xpert	+	+	ND	+2 days	Early detection	TP
		Culture	−	+	ND			
2	M/80	Xpert	+	+	ND	+3 days	Early detection	TP
		Culture	−	+	ND			
3	M/71	Xpert	−	+	+	+5 days	Early detection	TP
		Culture	−	−	+			
4	F/75	Xpert	+	+	ND	+15 days	Early detection	TP
		Culture	−	−	+			
5	M/90	Xpert	+	ND	ND	+15 days	Early detection	TP
		Culture	−	+	ND			
6	F/93	Xpert	+	+	ND	−1 day	Previous infection	FP
		Culture	+	−	ND			
7	M/74	Xpert	+	+	+	−4 days	Previous infection	FP
		Culture	+	+	−			
8	M/83	Xpert	NT	+	ND	−6 days	Previous infection	FP
		Culture	+	−	ND			
9	M/80	Xpert	+	+	+	−7 days	Previous infection	FP
		Culture	+	+	−			

a Subtraction of culture-positive day from Xpert-positive day. Positive and negative values represent early detection and previous infection, respectively. Calculation for early detected cases used the date of the first positive result, and previously infected cases used the date of the last positive result.

b Meaning of the discordant Xpert assay result based on interval between 2 assays for positive results.

c Our interpretation of the Xpert result.

Early detections were considered as TP according to our design. Xpert, Xpert MRSA assay; NSCLC, non-small cell lung cancer; ND, not done; TP, true-positive; FP, false-positive; M, male; F, female.
